# General synovitis score and immunologic synovitis score reflect clinical disease activity in patients with advanced stage rheumatoid arthritis

**DOI:** 10.1038/s41598-019-44895-9

**Published:** 2019-06-11

**Authors:** Tobias Schmidt, Aurélie Najm, Haider Mussawy, Rolf Burghardt, Nicola Oehler, Veit Krenn, Wolfgang Rüther, Andreas Niemeier

**Affiliations:** 1 Department of Orthopaedics, University Medical Center Hamburg-Eppendorf, Martinistraße 52, 20246 Hamburg, Germany; 2 Rheumatology Unit, Nantes University Hospital, 44093 Nantes, France; 3 INSERM UMR1238, Nantes University, 44093 Nantes, France; 4 Department of Pathology, Institute of Pathology, Max-Planck-Straße 18, 54296 Trier, Germany; 5 Institute of Osteology and Biomechanics IOBM, University Medical Center Hamburg-Eppendorf, Martinistraße 52, 20246 Hamburg, Germany

**Keywords:** Medical research, Rheumatoid arthritis

## Abstract

The purpose of this study was to investigate the relationship between clinical disease activity in patients with advanced stage rheumatoid arthritis (RA) on treatment with Disease Modifying Antirheumatic Drugs (DMARDs) and histopathological scores of synovial inflammation. To this end, synovial biopsies of 62 RA patients who underwent surgery for either synovectomy or total joint arthroplasty were assessed by a general synovitis score (GSS) and an immunologic synovitis score (IMSYC). The clinical disease activity index (CDAI) was significantly correlated with both the GSS and the IMSYC (r = 0.65, p = <0.001, r = 0.68, p = <0.001). Compared to patients with moderate and high disease activity, there was a significantly lower expression of T cell (CD3), B cell (CD20) and neutrophil (CD15) markers in synovial tissue of patients with low activity, but similar expression of the macrophage marker CD68. Subgroup analyses revealed no differences between small and large joints, seropositive and seronegative RA and patients with or without prednisolone treatment. However, we found a significantly stronger correlation of CDAI with IMSYC in patients undergoing arthroplasty (r = 0.82) than in patients undergoing synovectomy (r = 0.55). In addition, there was a stronger correlation of CDAI with GSS in patients treated with methotrexate (r = 0.86) than in patients with TNFα blockade (r = 0.55). In summary, the present study demonstrates that the histopathological scores GSS and IMSYC in general reflect clinical disease activity in patients with advanced stage rheumatoid arthritis, but that there is some heterogeneity between subgroups of patients within the cohort. In the future, molecular characterization of synovial inflammatory cell populations, including plasma cell infiltrates, will help to further defined clinically important subtypes of RA and treatment response.

## Introduction

Rheumatoid arthritis (RA) is the most prevalent autoimmune arthritis, affecting approximately 1% of the population. If left untreated, chronic synovial membrane inflammation in RA causes progressive joint destruction. While diagnosis is established by clinical symptoms and laboratory tests for citrullinated peptide (CCP) antibodies or rheumatoid factor (RF), the clinical disease activity is monitored by standardized scores such as Disease Activity Score 28 (DAS-28), clinical disease activity index (CDAI) and simplified disease activity index (SDAI). The goal of treatment is to maintain low disease activity or to induce remission^1^, which can be accomplished by the use of conventional synthetic disease- modifiying anti-rheumatic drugs (DMARDs) such as methotrexate or by biological DMARDs that are highly effective in inducing remission^2^. The most frequently used biological class of drugs are TNFα inhibitors. Nevertheless, 20–30% of RA patients are not responding to TNFα blockade, resulting in persistent synovitis^3^. Most of these non- responders display persistent swelling of multiple joints and tendon sheaths^4^.

The histopathological General Synovitis Score (GSS) has been developed in order to distinguish inflammatory arthritis from non-inflammatory arthritis^5^. This score considers three components of synovitis: lining layer hyperplasia, activation of resident cells (stroma) and inflammatory infiltrate. All of these components are graded semi-quantitatively from 0 to 3 and the total score ranges from 0 to 9^5,6^. High-grade synovitis is defined by a score higher than 4. Along similar lines, but much more sophisticated on a molecular level, recently published gene expression analyses of RA synovial tissue revealed low inflammatory and high inflammatory subtypes that demonstrate a functional correlation with markers of systemic inflammation and peripheral T-cells^7,8^. In addition, the immunologic synovitis (IMSYC) score has been reported to further improve characterization of synovitis in RA^9^.

Although widely used in clinical routine and often cited, it is not even clear whether and how the general synovitis score GSS reflects clinical disease activity in medically treated RA patients. Therefore, the purpose of this study was to assess the correlation of the synovitis scores GSS and IMSYC with the clinical disease activity of 62 DMARD treated late stage RA patients.

## Methods

### Patients

Synovial biopsies were taken intraoperatively from 62 consecutive patients with rheumatoid arthritis that underwent surgery for either isolated synovectomy (n = 30 patients) or total joint replacement plus synovectomy or arthrodesis (n = 32 patients). Synovial biopsies were collected from all joints that were operated on, including metacarpophalangeal joints (n = 12), knee joints (n = 12), hip joints (n = 10), glenohumeral joints (n = 6), cubital joints (n = 5), metatarsophalangeal joints (n = 5), proximal interphalangeal joints (n = 5), upper ankle joints (n = 3), talonavicular joints (n = 2), carpometacarpal joint (n = 1) and tarsometatarsal joint (n = 1). Diagnosis of RA was made according to the ACR criteria^10^. Inclusion criterea were ongoing medication prior to surgery for at least six months with either methotrexate or a TNFα inhibitor. Patients with different DMARD medication were excluded. Thirtythree patients additionally received low dose prednisolone therapy (<7.5 mg/day) within 12 month prior to surgery. Disease activity was evaluated using DAS28-CRP score (four variables, CRP-based), CDAI and SDAI as described previously^11,12^ at the time point of surgery. The study was conducted in accordance with the Declaration of Helsinki and approved by the Ethics Committee of University Medical Center Hamburg-Eppendorf (PV5008). All patients gave written consent to participate in the study.

### Synovial biopsies

Fresh synovial biopsy tissue samples were fixed overnight in 4% formalin buffer at pH 7.0 and embedded in paraffin for histologic and immunohistochemical analyses. Serial histologic sections were stained with hematoxylin and eosin. Immunostaining was performed on an automated staining system (BenchmarkXT, IHC Slide Stainer der Marke Roche, Ventana Medical Solutions). The following antibodies were used: anti-CD3 (Klon: 2GV6; 7341, Roche, Basel, Schweiz), anti-CD20 (Klon: L26; 1044, Roche, Basel, Schweiz), anti-CD 15 (Klon: MMA; 2599, Roche, Basel, Schweiz), anti-CD68 (Klon: KP-1; 1036, Roche, Basel, Schweiz), and Ki67 (Klon: 30-9; 3467, Roche, Basel, Schweiz). GSS was performed as described previously^5,13^. The quantitative evaluation of immune cell population was performed by the principle of maximum focal infiltration (focus) together with a manual count of this single focal point (1.3 mm^2^). All slides were stained and scored in a blinded fashion. Slides were scored by two reviewers independently for GSS score and for each immunostaining using a semi-quantitative 4 scale score (0–3) with following range of the manual count; 0 = 1–9 (no infiltrate), 1 = 10–99 (mild infiltrate), 2 = 100–999 (moderate infiltrate) and 3 = >1000 (severe infiltrate). The features were added for a total score out of 24 (GSS 0–9 points and 0–3 for each of the immunostainings).

### Statistics

Statistical analyses were performed using GraphPad Prism 5.0 for Windows (GraphPad Software, Inc., La Jolla,CA, USA). Normal distribution of the data was tested with the Kolmogorov–Smirnov test. While parametric data were assessed for statistical correlation using Pearson’s correlation, nonparametric data were assessed using Spearman’s rho. A Fisher’s r-to-z transformation test was performed to test for potential differences between correlations. To compare the study groups in terms of continuous variables, the unpaired two-sided *t*-test and ANOVA were used with the normally distributed data while the Mann−Whitney U test and Kruskal-Wallis test were used with the non-normally distributed data. P values of <0.05 were considered as statistically significant.

## Results

### Demographic and disease characteristics of the study cohort

The study cohort was composed of 62 consecutive patients with RA who met the inclusion criteria with varying clinical disease activity. The median for DAS28-CRP, CDAI and SDAI was 2.39, 5.0 and 5.25, respectively. The mean age of patients was 61.8 years (range, 22–88 years). The majority of patients (69.4%) were female and the mean duration of disease was 13.1 years. All patients had been diagnosed with RA for at least two year prior to surgery. Hence, the cohort consisted mostly of late-stage RA patients. Synovial biopsies were collected from different joints as shown in Table 1, which also summarizes the main characteristics of the study cohort. Supplemental Table 1 provides a complete list of all individual clinical, histological and immunohistochemical data.

**Table 1:** Demographic and disease specific characteristics of RA patients.

	Mean/Median(±SD/IQR)or n (%)
n	62
Mean age, (years)	61.8 (13.4)
Sex (no. of female, %)	43/62 (69.4)
Mean disease Duration (years)	13.1 (8.7)
Seropositive (CCP+)	50/62 (80.6%)
**Surgery**	
Synovectomy	30/62 (48.4)
Joint Replacement/Arthrodesis	32/62 (51.6)
**Medical treatment**	
Methotrexate	18/62 (29.0%)
Etanercept	22/62 (35.5%)
Adalimumab	12/62 (19.3%)
Certolizumab	4/62 (6.4%)
Infliximab	4/62 (6.4%)
Golimumab	2/62 (3.2%)
Prednisolon	33/62 (53.2%)
**Synovial biopsies (joints)**	
Metacarpophalangeal joint	12 (19.3%)
Knee joint	12 (19.3%)
Hip joint	10 (16.1%)
Glenohumeral joint	6 (9.6%)
Cubital joint	5 (8.1%)
Metatarsophalangeal joint	5 (8.1%)
Proximal interphalangeal joint	5 (8.1%)
Upper ankle joint	3 (4.8%)
Talonavicular joint	2 (3.2%)
Carpometacarpal joint	1 (1.6%)
Tarsometatarsal joint	1 (1.6%)
**Laboratory values**	
CRP (mg/l) median	4 (1–10)

### General synovitis score and immunologic synovitis reflect the clinical disease activity

**Figure 1:**
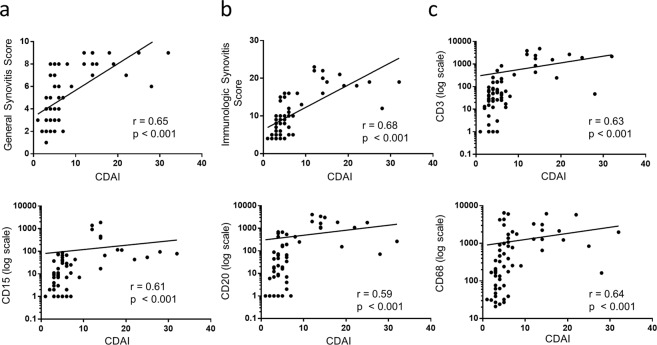
GSS, IMSYC, CD3, CD15, CD20 and CD68 reflect the clinical disease activity in patients with rheumatoid arthritis. Clinical disease activity index (CDAI) was measured at time point of surgery and correlated with (**a**) general synovitis score (**b**) immunologic synovitis score and (**c**) CD3, CD15, CD20 and CD68 expression in 62 RA patients. P values < 0.05 were considered statistically significant.

**Figure 2:**
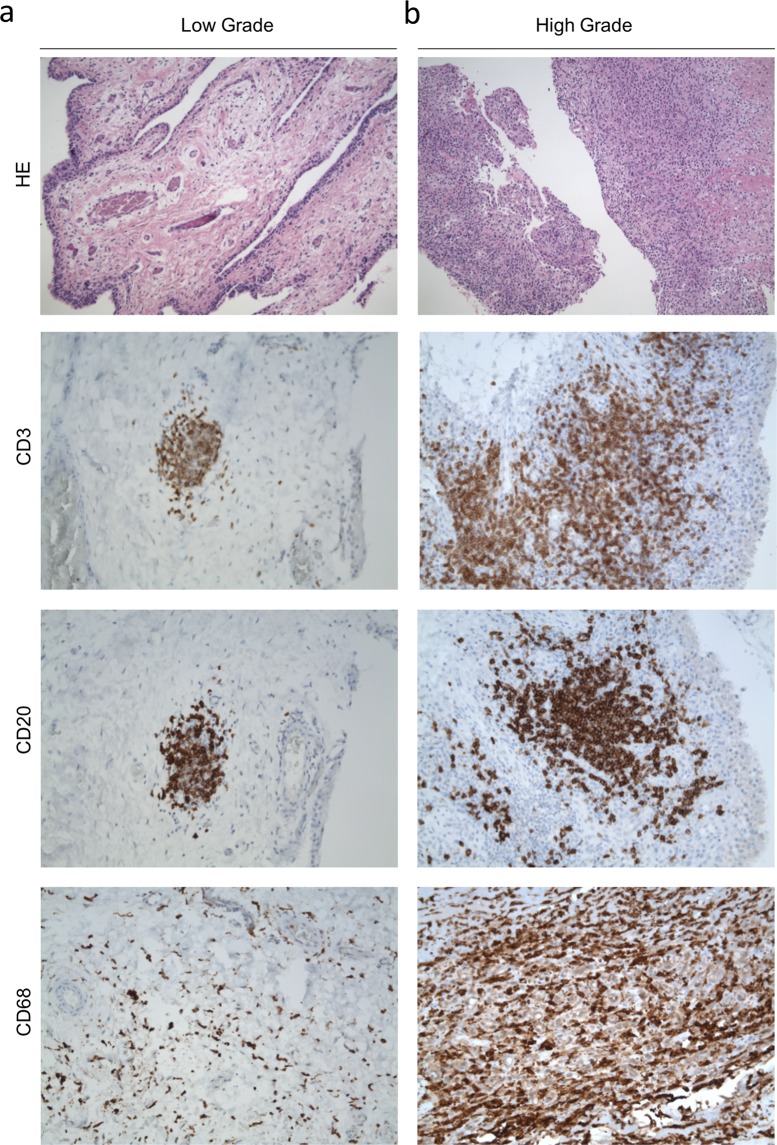
Examples of low and high grade inflammation in H&E staining and for T cells (CD3), B cells (CD20) and macrophages (CD68).

The clinical scores DAS28-CRP, CDAI and SDAI were significantly correlated with each other in the study cohort (Supplemental Fig. 1). For the sake of simplicity and readability of the manuscript, we therefore used only the CDAI as a representative score for all further analyses. We first assessed the relationship of clinical disease activity as measured by CDAI with the GSS, IMSYC, CD3, CD15, CD20 and CD68 scores (Fig. 1). Here, we found the highest correlation with the IMSYC (r = 0.68, p < 0.001) followed by GSS (r = 0.65, p < 0.001), CD68 (r = 0.64, p < 0.001), CD3 (r = 0.66, p < 0.001), CD15 (r = 0.61, p < 0.001) and CD20 (r = 0.59, p < 0.001). Figure 2 demonstrates exemplary pictures of synovial samples with low vs. high grade infiltration in H&E staining, as well as CD3, CD20 and CD68 staining.

### Correlation of CDAI with synovial GSS and IMSYC is stronger in patients with methotrexate than in patients with TNFα blocking therapy and stronger in patients undergoing arthroplasty than in patients undergoing synovectomy

**Figure 3:**
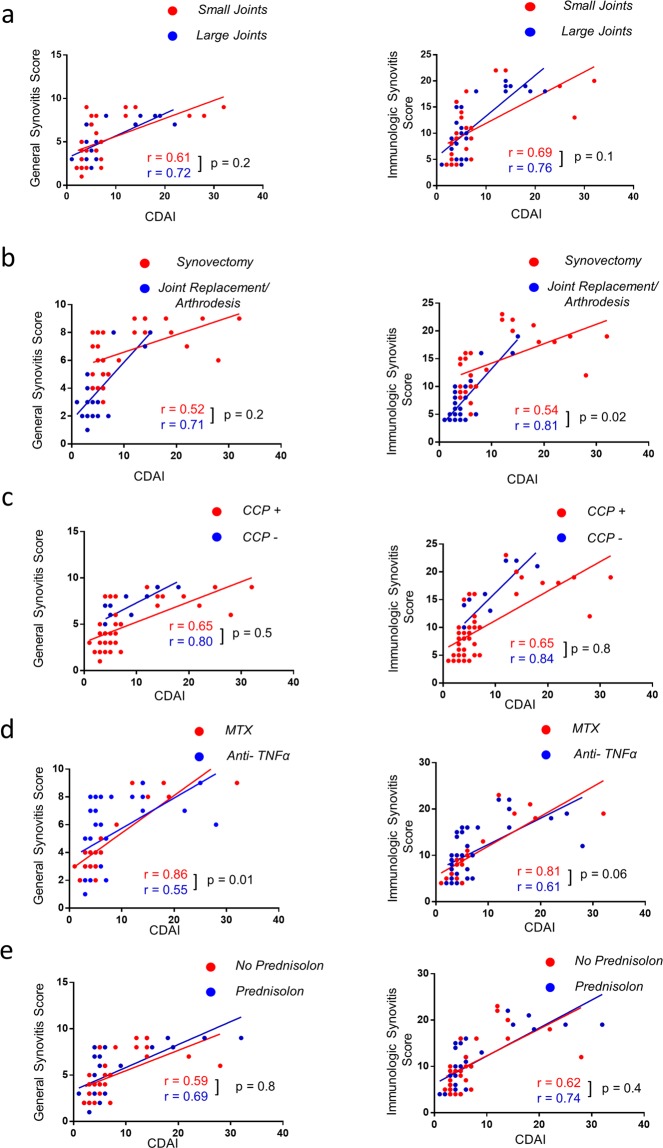
Correlation of clinical disease activity with synovial GSS and IMSYC is higher in patients with methotrexate than in patients with TNFα blocking therapy. Clinical disease activity index (CDAI) was correlated with (**a**) the GSS and the IMSYC separately in patients with small or large joints (red dots and red regression line), (**b**) in patients with indication for joint replacement/arthrodesis or synovectomy (**c**) in patients seropositive for CCP or seronegative (**d**) in patients with ongoing anti-TNFα therapy and (**e**) in patients with prednisolone therapy within last 12 month or no prednisolone therapy. p values < 0.05 were considered statistically significant.

We next analyzed the data with regard to important clinical factors by dividing the cohort into subgroups. We first compared patients with synovial tissue from large joints (shoulder, elbow, hip and knee) to small joints (metacarpophalangeal, metatarsophalangeal, proximal interphalangeal, upper ankle, talonavicular, carpometacarpal and tarsometatarsal). Here, the correlation in the group of large joints was slightly stronger both for GSS (r = 0.72 vs. r = 0.61) and IMSYC (r = 0.76 vs. r = 0.69) (Fig. 3a). In contrast, when stratifying according to the procedure performed and thus comparing patients that underwent synovectomy to patients that underwent arthroplasty, there was a significant difference. The correlation of IMSYC with CDAI was stronger in the group with joint replacement or arthrodesis (r = 0.81 vs. r = 0.54) than in the synovectomy group. No differences were observed between seropositive and seronegative patients (Fig. 3c). However, when comparing the group with MTX therapy to the group with TNFα blocking therapy we found a significant stronger correlation in the group with MTX therapy for the GSS (r = 0.86 vs. r = 0.55) and a non-significant difference for the IMSYC score (r = 0.81 vs. r = 0.61). On the contrary, correlation of GSS and IMSYC with CDAI of patients that had received prednisolone therapy within 12 month prior to surgery did not differ from patients without prednisolone therapy.

### Patients with low disease activity display low infiltration of T cells, B cells and neutrophils but pronounced macrophage infiltration

**Figure 4:**
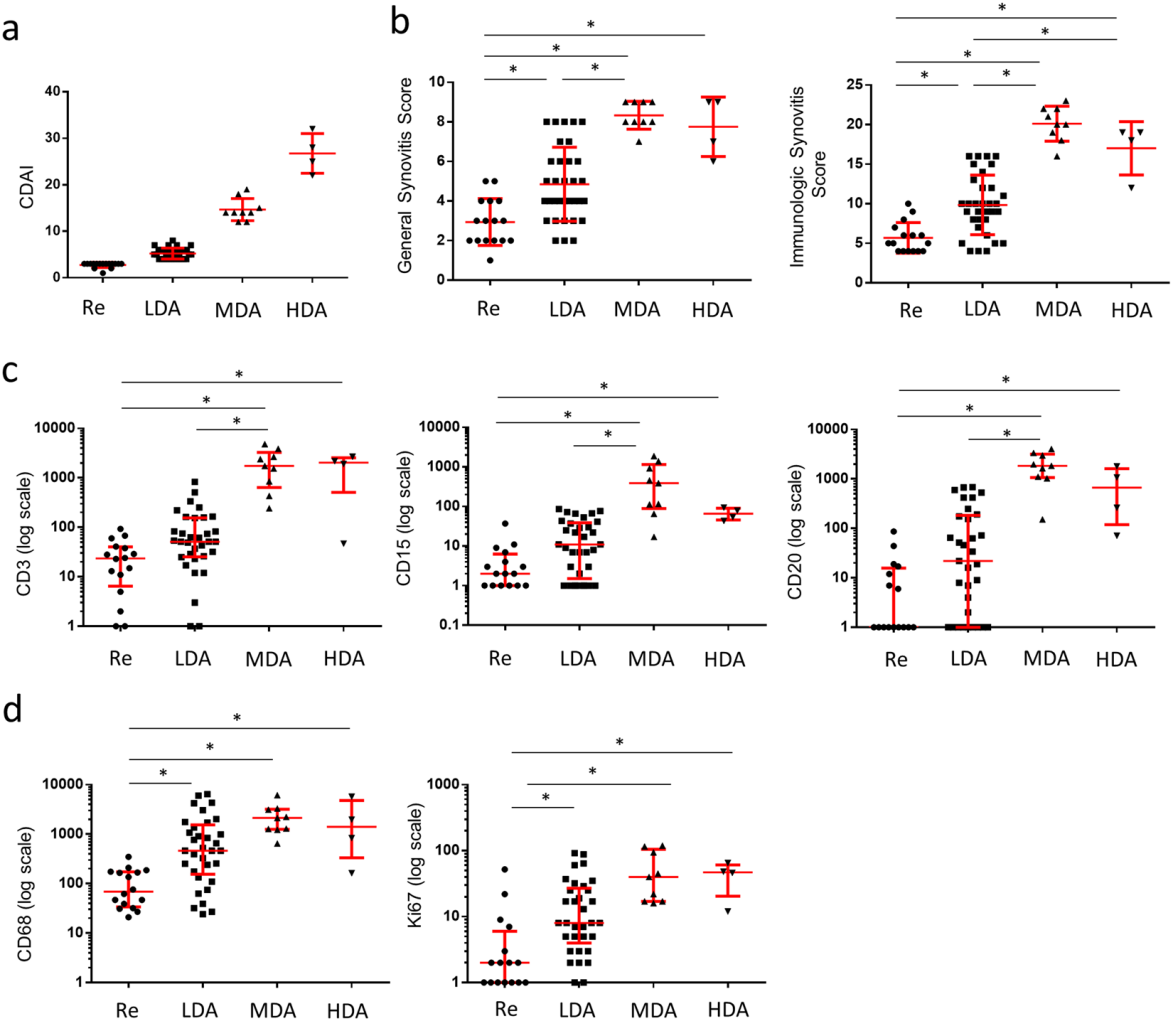
Patients with low disease activity display low infiltration of T cells, B cells and neutrophils but high macrophages infiltration. The RA Patients cohort was divided according to clinical disease activity index (CDAI) in 4 groups (**a**) with Remission (Re) ≤2.8, low disease activity (LDA) ≤10, moderate disease activity (MDA) ≤22, and high disease activity (HAD) >22 as previously proposed^12^. The 4 groups were compared with (**b**) GSS and IMSYC and (**c**) CD 3, CD15, CD20 and (**d**) CD68 and Ki67 expression. *p values < 0.05 were considered statistically significant.

To identify potential differences in the infiltration of immune cells in the synovial membrane in patients with different clinical disease activity, we divided the cohort in four groups (1) remission (2) low activity (3) moderate activity and (4) high activity according to CDAI (Fig. 4a). Here we used the following thresholds as previously proposed^14^; Remission ≤2.8, low disease activity ≤10, moderate disease activity ≤22, high disease activity >22. As expected, both GSS and IMSYS were significantly lower in the group of patients in remission and with low disease activity (Fig. 4b) with no differences between moderate and high disease activity. Similarly, we found significant differences in the expression of CD3, CD20 and CD15 between moderate and high disease activity groups (Fig. 4c). In contrast, no significant differences were observed between patients with low disease activity and moderate/high disease activity in the expression of CD68 (Fig. 4d). We also compared the specific synovial membrane immune profile of outlier (low disease activity and GSS >5 and IMSYC >11) to patients with high disease activity and high GSS >5 and IMSYC >11. Here we found significantly differences in the expression of CD3, CD15, CD20 but no differences in the expression of CD68 and Ki67 (Supplemental Fig. 2a–e.)

## Discussion

Here we demonstrate that in late-stage RA patients under MTX and TNF alpha blockade, both GSS and IMSYC significantly correlate with the clinical disease activity.

Prior to this study, GSS has been established as an effective diagnostic tool to distinguish rheumatic and degenerative joint diseases, in particular osteoarthritis and rheumatoid arthritis. In a large study of 559 synovial samples, the overall sensitivity and specificity of the GSS for diagnosis of a rheumatic disease was reported to be 61.7% and 96.1%^5^. We recently demonstrated that the IMSYC represents a more functional synovitis evaluation with a better sensitivity and specificity than the GSS^9^. Despite its diagnostic value and its acceptance in clinical routine, the GSS has not been correlated with clinical disease activity of rheumatoid patients on anti-rheumatic medication in larger patient cohorts. Today, most patients are treated effectively with DMARDs, with varying success. Therefore, the current study was designed to investigate the correlation of the synovitis scores GSS and IMSYC with clinical disease activity in patients with late-stage RA on DMARD treatment.

An interesting finding of the current study is that the correlation in patients with anti-TNFα therapy was lower than in patients with methotrexate treatment. Similar to this, it has been reported that there is more variability in clinical and radiographic outcomes in patients treated with biologicals than with synthetic conventional DMARDs^15,16^. Another explanation may be that localized treatment failure with otherwise good treatment response occurs more often in patients with anti TNFα therapy compared to patients with methotrexate therapy. Low dose methotrexate as used in RA has been demonstrated to be effective to suppress both T and B cells^17^, which can be due to a direct effect by preventing lymphotoxin synthesis by folate antagonism^18^. Moreover, as methotrexate increases adenosine levels, an inhibitory effect on neutrophils has been suggested^19^. The effects of methotrexate on TNFα production is less clear. While some studies suggest little effect^20^, others have reported reduction in TNFα levels following methotrexate treatment of synovial biopsies samples from RA patients^21^. TNFα antagonists exert their immunosuppressive effects by direct suppression of TNFα which plays a central role in rheumatoid arthritis through the activation of cytokine and chemokine production, elevated expression of endothelial-cell adhesion molecules and promotion of angiogenesis^22^. TNFα also acts as a positive feedback signal to further promote development and survival of macrophages and blockade of TNF-α signaling in macrophages can promote reduction of macrophages^23^. However, the effect of TNFα antagonist on B cells are conflicting and likely more indirectly compared to methotrexate^24^.

In summary, the higher correlation of clinical disease activity with synovial inflammation and immunological scores in patients on methotrexate treatment might in part be explained by the broader immunosuppressive effect of methotrexate compared to the more specific effects of TNFα antagonist on inflammatory cells. Certainly, further studies are required to confirm our observation and to compare the immune profile of different joints in relation to therapy.

To exclude potential selection bias related to the clinical selection, surgical indication and therapy of the RA patients, we performed different sub-group analyses. Subgroup analysis revealed that in both groups GSS and IMSYC were significantly correlated with CDAI with an even stronger correlation in the group receiving joint replacement or arthrodesis, indicating that this subgroup represents a more homogenous cohort than the synovectomy group. However, these results need to be interpreted with caution as most of the patients undergoing joint replacement or arthrodesis had low GSS, IMSYC and CDAI scores, turning some of the indistinguishable from OA-like low grade synovitis simply by GSS or IMSYC scoring^9^. Nevertheless, in general, it is unlikely that the underlying pathology equals that of OA since recent studies suggest that RA patients undergoing joint replacement frequently suffer from flares independent of the respective anti-rheumatic medication^25^.

The fact that further comparisons of distinct subgroups, i.e. large vs. small joints, seropositive vs seronegative RA and prednisolone vs no prednisolone use, did not reveal any differences suggests that future studies should aim more detailed molecular analyses of synovial inflammation in different joints and under specific conditions in order to expand our knowledge about subtypes of synovial inflammation.

One interesting finding of this study is that macrophage infiltration - unlike T cell, B cell and neutrophil infiltration - did not significantly differ between patients with low clinical disease activity and patients with moderate or high disease activity. Furthermore, when we compared the outliers in this study who showed low disease activity but high GSS and IMSYC scores, we found significantly lower T cell, B cell and neutrophil infiltration but similar macrophages infiltration. Interestingly, all of the ten outliers were only affected at one specific joint and nine of these ten patients were using TNFα antagonists. This suggests that some patients with refractory monosynovitits generally react to treatment by effectively suppressing T cells, B cells and neutrophils numbers but fail to prevent macrophages infiltration in the affected joint. Based on these findings, future studies will help to further delineate the biological basis of this particular local synovial reaction and thereby may open perspectives the development of tissue driven therapy. It is important to mention that we used CD68 as a global marker of macrophages. It would certainly be of clinical relevance to differentiate between pro-inflammatory M1 and anti-inflammatory M2 type macrophages in this context^26^. This could help to develop more specific biomarkers for prognosis and therapeutic decision making. Recently, blockade of granulocyte-macrophage colony-stimulating factor (GM-CSF) has been shown to effectively suppress myeloid cell activity in rheumatoid arthritis patients and thus may be also an interesting treatment option for patients with general low disease activity but persistent strong macrophage infiltration in the affected joint^27,28^.

While the present study aimed to correlate established synovitis scores with the clinical disease activity, the addition of more specific immunostaining could potentially help to characterize distinct profiles of RA subtypes^29^^,^^30^. Plasma cells are terminally differentiated B cells that synthesize antibodies to preserve humoral immunity. By the production of pathogenic antibodies, plasma cells are involved in the pathogenesis of many autoimmune diseases. In this context, plasma cells have been identified as an important marker of rheumatic disorder in distinction to osteoarthritis and have also been reported to differentiate early from later stages of rheumatoid arthritis^7^. More specifically, the histologic features that strongly define high inflammatory subtypes in RA were shown to include 3 plasma cell features: binucleated plasma cells, percentages of plasma cells, and Russell bodies^7^. A detailed analysis of plasma cell distribution in the synovial membrane in relation to specific therapy could therefore potentially help to identify mechanisms of therapy resistance. It has also recently been reported that specific subtypes of T cells infiltrate synovial tissue and strongly interact with specific B cells within the inflamed tissue. In addition functionally distinct fibroblasts with expression of the proteins THY1 and cadherin-11, but lacking CD34 expression^31^ have been identified very recently. Similarly, mast cell infiltration has been demonstrated to be associated with local and systemic inflammation, autoantibody positivity and high disease activity^32^. Hence, in future studies, staining of further mast cells, fibroblast-, T- and B-cell subpopulations will help to identify synovitis-subtypes of RA that differ in response to treatment^8^. This may pave the way for prognostic synovial analysis as a tool to predict treatment response in the future.

The identification of specific immune cell subtypes in RA, represents a rapidly emerging filed in RA research. Most recently, three distinct synovial subtypes have been described: a high inflammatory subtype, a low inflammatory subtype and a mixed subtype^7^. In this study, the authors applied machine learning to histologic features with gene expression subtypes serving as labels and thereby developed a specific algorithm for the scoring of histological features.

We propose that for future studies both approaches, direct immunostaining such as IMSYC scores with more detailed subtype staining and machine learning with integration of RNA data could help to better understand the characteristics of certain RA subtypes in relation to therapy.

There are limitations to the present study. Firstly, our patient cohort was a created from consecutive RA patients in an orthopaedic center with special focus and recognized expertise in orthopaedic rheumatology and thus would be considered a tertiary care center. This fact may imply a selection bias in the surgical cases, which may not be necessarily representative for the entire RA population. Here we analyzed late stages of the disease. In early stages of RA the correlation of clinical disease activity with synovial inflammation and immune cell infiltration might differ significantly and will have to be investigated separately. Secondly, GSS and IMSYC have high diagnostic value in RA and seem to reflect the clinical disease activity in late stage RA. However, the scores are only basic tool of synovial assessment and needs to be expanded further in the future by the addition of distinct cell subtype markers for plasma cells, mast cells or specific T cells as biomarkers for both prognosis and therapeutic decision. Taken together, we consider both limitations as relevant factors that need to be recognized in the design of future studies, but think that neither profoundly affects the main findings and conclusions from the present study.

In summary, here we demonstrate that the GSS and the immunologic synovitis score generally reflect the disease activity in RA in medically treated patients. The fact that correlations are lower in patients treated with TNFα blockade should prompt further investigations in the heterogeneity of biological response of joints with persistent synovitis despite TNF blockade. Future studies in this regard will most probably benefit from new methods including molecular synovial cell profiling.

### Compliance with ethical standards

Written informed consent was obtained from participants for research studies and presented data. All research studies have been performed according to the rules of the local ethics committee of the University Medical Centre Hamburg-Eppendorf, Germany. The local ethics committee of the University Medical Center Hamburg-Eppendorf approved this study (PV5008).

## Supplementary information

Supplementary Information

Supplemental Table 1

## References

[1] Cader MZ (2011). Performance of the 2010 ACR/EULAR criteria for rheumatoid arthritis: comparison with 1987 ACR criteria in a very early synovitis cohort. Ann Rheum Dis.

[2] Weinblatt ME (2003). Adalimumab, a fully human anti-tumor necrosis factor alpha monoclonal antibody, for the treatment of rheumatoid arthritis in patients taking concomitant methotrexate: the ARMADA trial. Arthritis Rheum.

[3] Vincent FB (2013). Antidrug antibodies (ADAb) to tumour necrosis factor (TNF)-specific neutralising agents in chronic inflammatory diseases: a real issue, a clinical perspective. Ann Rheum Dis.

[4] Badot V (2009). Gene expression profiling in the synovium identifies a predictive signature of absence of response to adalimumab therapy in rheumatoid arthritis. Arthritis Res Ther.

[5] Krenn V (2006). Synovitis score: discrimination between chronic low-grade and high-grade synovitis. Histopathology.

[6] Slansky E (2010). Quantitative determination of the diagnostic accuracy of the synovitis score and its components. Histopathology.

[7] Orange DE (2018). Identification of Three Rheumatoid Arthritis Disease Subtypes by Machine Learning Integration of Synovial Histologic Features and RNA Sequencing Data. *Arthritis &*. Rheumatology.

[8] Rao DA (2017). Pathologically expanded peripheral T helper cell subset drives B cells in rheumatoid arthritis. Nature.

[9] Najm, A. *et al*. IMSYC IMmunologic SYnovitis sCore: A New Score for Synovial Membrane Characterization In Inflammatory And Non-Inflammatory Arthritis. *Joint Bone Spine* (2018). 10.1016/j.jbspin.2018.04.004 29709696

[10] Aletaha D (2010). Rheumatoid arthritis classification criteria: an American College of Rheumatology/European League Against Rheumatism collaborative initiative. Arthritis Rheum.

[11] Makinen H, Hannonen P, Sokka T (2006). Definitions of remission for rheumatoid arthritis and review of selected clinical cohorts and randomised clinical trials for the rate of remission. Clin Exp Rheumatol.

[12] Aletaha D (2005). Acute phase reactants add little to composite disease activity indices for rheumatoid arthritis: validation of a clinical activity score. Arthritis Res Ther.

[13] Kolbel B (2015). CD15 focus score for diagnostics of periprosthetic joint infections: Neutrophilic granulocytes quantification mode and the development of morphometric software (CD15 quantifier). Z Rheumatol.

[14] Aletaha D, Smolen J (2005). The Simplified Disease Activity Index (SDAI) and the Clinical Disease Activity Index (CDAI): a review of their usefulness and validity in rheumatoid arthritis. Clin Exp Rheumatol.

[15] Emery P (2009). Less radiographic progression with adalimumab plus methotrexate versus methotrexate monotherapy across the spectrum of clinical response in early rheumatoid arthritis. J Rheumatol.

[16] Smolen JS (2005). Evidence of radiographic benefit of treatment with infliximab plus methotrexate in rheumatoid arthritis patients who had no clinical improvement: a detailed subanalysis of data from the anti-tumor necrosis factor trial in rheumatoid arthritis with concomitant therapy study. Arthritis Rheum.

[17] Wascher TC (1994). Cell-type specific response of peripheral blood lymphocytes to methotrexate in the treatment of rheumatoid arthritis. Clin Investig.

[18] Chan ESL, Cronstein BN (2010). Methotrexate—how does it really work?. Nature Reviews Rheumatology.

[19] Cronstein BN (1992). Neutrophil adherence to endothelium is enhanced via adenosine A1 receptors and inhibited via adenosine A2 receptors. J Immunol.

[20] Williams AS, Punn YL, Amos N, Cooper AM, Williams BD (1995). The effect of liposomally conjugated methotrexate upon mediator release from human peripheral blood monocytes. Br J Rheumatol.

[21] Dolhain RJ (1998). Methotrexate reduces inflammatory cell numbers, expression of monokines and of adhesion molecules in synovial tissue of patients with rheumatoid arthritis. Br J Rheumatol.

[22] Kalliolias GD, Ivashkiv LB (2015). TNF biology, pathogenic mechanisms and emerging therapeutic strategies. Nature Reviews Rheumatology.

[23] Catrina AI (2005). Evidence that anti-tumor necrosis factor therapy with both etanercept and infliximab induces apoptosis in macrophages, but not lymphocytes, in rheumatoid arthritis joints: extended report. Arthritis Rheum.

[24] Glaesener S (2014). Distinct effects of methotrexate and etanercept on the B cell compartment in patients with juvenile idiopathic arthritis. Arthritis Rheumatol.

[25] Goodman SM (2018). Flares in Patients with Rheumatoid Arthritis after Total Hip and Total Knee Arthroplasty: Rates, Characteristics, and Risk Factors. J Rheumatol.

[26] Udalova IA, Mantovani A, Feldmann M (2016). Macrophage heterogeneity in the context of rheumatoid arthritis. Nat Rev Rheumatol.

[27] Weinblatt ME (2018). A Randomized Phase IIb Study of Mavrilimumab and Golimumab in Rheumatoid Arthritis. Arthritis Rheumatol.

[28] Guo X (2018). Blockade of GM-CSF pathway induced sustained suppression of myeloid and T cell activities in rheumatoid arthritis. Rheumatology (Oxford).

[29] Smolen JS, Aletaha D, Redlich K (2012). The pathogenesis of rheumatoid arthritis: new insights from old clinical data?. Nat Rev Rheumatol.

[30] Boissier MC (2011). Cell and cytokine imbalances in rheumatoid synovitis. Joint Bone Spine.

[31] Mizoguchi F (2018). Functionally distinct disease-associated fibroblast subsets in rheumatoid arthritis. Nat Commun.

[32] Rivellese, F. *et al*. Mast cells in early rheumatoid arthritis associate with disease severity and support B cell autoantibody production. *Ann Rheum Dis* (2018). 10.1136/annrheumdis-2018-213418 30127058

