# Supplementary material for: General synovitis score and immunologic synovitis score reflect clinical disease activity in patients with advanced stage rheumatoid arthritis
Source: Sci Rep. 2019 Jun 11;9:8448. doi: 10.1038/s41598-019-44895-9 (PMC6560084; doi:10.1038/s41598-019-44895-9)
Supplement: Supplementary file 1 — Supplementary Information [file 41598_2019_44895_MOESM1_ESM.pdf]

Supplemental Information:

General synovitis score and immunologic synovitis score reflect clinical disease activity in patients with advanced stage rheumatoid arthritis

Tobias Schmidt[1,5], Aurélie Najm[2,3], Haider Mussawy[1], Rolf Burghardt[1], Nicola Oehler[1], Veit Krenn[4], Wolfgang Rüther[1] and Andreas Niemeier[1]

[1]Department of Orthopaedics, University Medical Center Hamburg-Eppendorf, Martinistraße 52, 20246 Hamburg, Germany

[2] Rheumatology Unit, Nantes University Hospital, 44093 Nantes, France.

3 INSERM UMR1238, Nantes University, 44093 Nantes, France

[4] Department of Pathology, Institute of Pathology, Max-Planck-Straße 18, 54296 Trier, Germany

[5]Institute of Osteology and Biomechanics IOBM, University Medical Center Hamburg-Eppendorf, Martinistraße 52, 20246 Hamburg, Germany

Supplemental Figure 1:

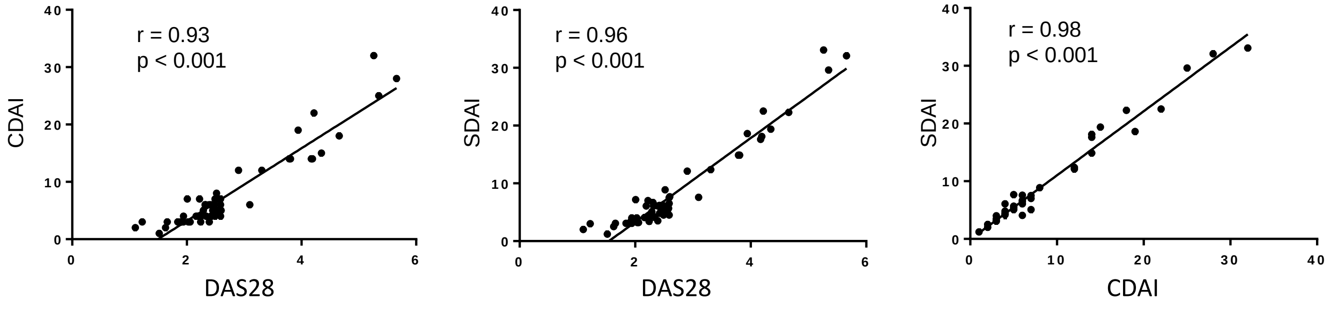

Supplemental Figure 1: Correlation of different clinical disease activity scores
Disease Activity Score 28 (DAS28), clinical disease activity index (CDAI) and Simplified Disease Activity Index (SDAI) were correlated with each other

Supplemental Figure 2:

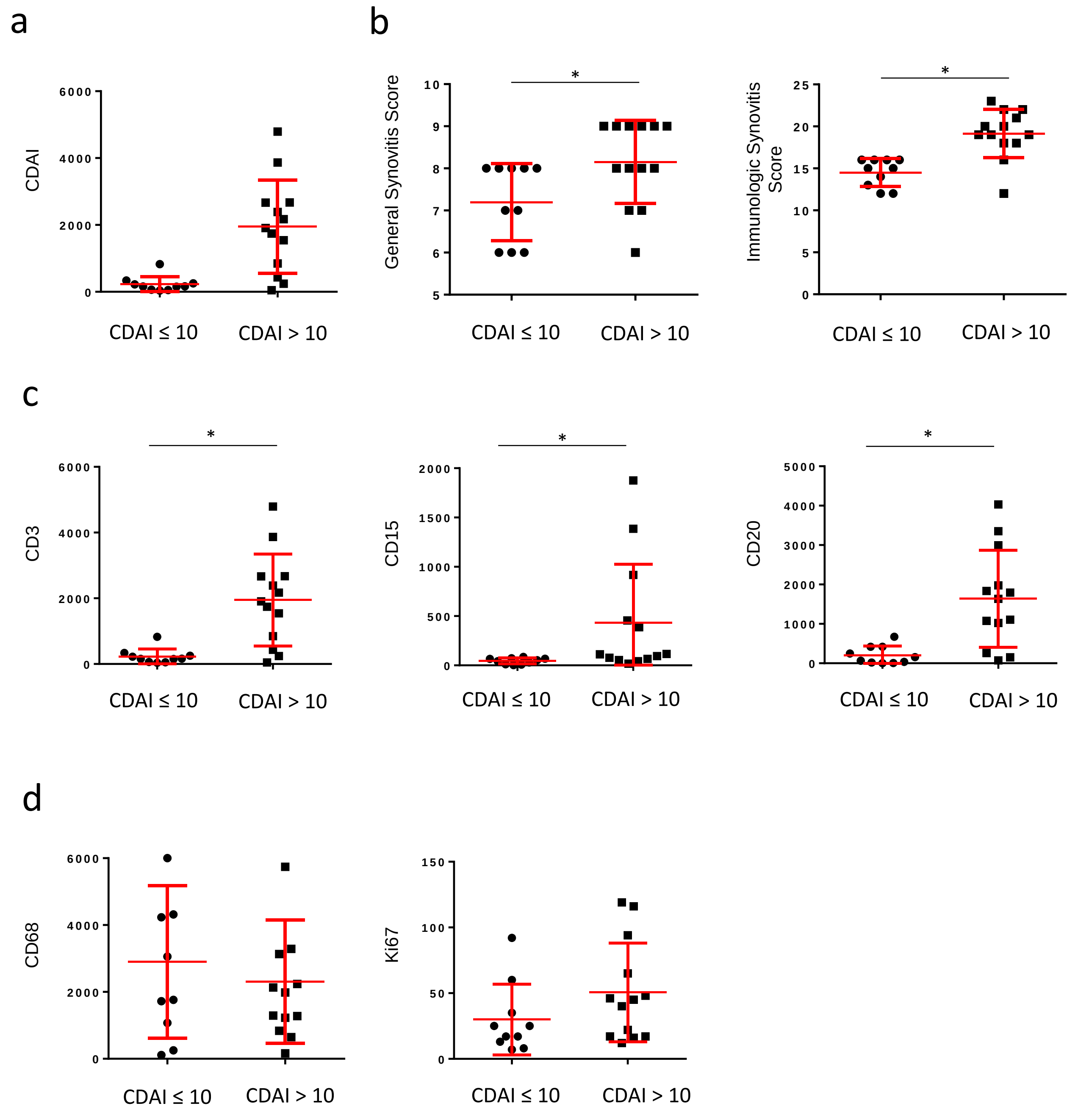

Supplemental Figure 2: *Patients with refractory monosynovitis display low infiltration of T cells, B cells and neutrophils but high macrophages infiltration*

Patients with low clinical disease activity (CDAI ≤ 10) and high synovial membrane inflammation (GSS > 5 and IMSYC >11) were compared to patients with moderate or high clinical disease activity (CDAI > 10) for (b) GSS and IMSYC and (c) CD 3, CD15, CD20 and (d) CD68 and Ki67 expression. * p values < 0.05 were considered statistically significant.
